# Seminal Vesicle Atrophy and Ejaculatory Dysfunction Secondary to Prostatic and Seminal Vesicle Abscesses

**DOI:** 10.7759/cureus.81917

**Published:** 2025-04-08

**Authors:** Shinya Miyazaki, Takashi Ueda, Masashi Tsujimoto, Jintetsu Soh, Osamu Ukimura

**Affiliations:** 1 Department of Urology, Kyoto Prefectural University of Medicine, Kyoto, JPN; 2 Department of Urology, Japanese Red Cross Society Kyoto Daini Hospital, Kyoto, JPN

**Keywords:** complication, ejaculatory dysfunction, prostate abscess, seminal vesicle abscess, urogenital atrophy

## Abstract

Seminal vesicle (SV) and prostatic abscesses are rare urological infections. However, their effect on sexual function remains unknown. In the present study, we encountered a case of SV atrophy and ejaculatory dysfunction (EjD) after conservative treatment. The patient was a 56-year-old man with fever who complained of pain during urination. He was diagnosed with SV and prostate abscesses and treated conservatively with antibiotics. One month after treatment, an EjD was observed, and magnetic resonance imaging revealed severe atrophy of the SV. Although various treatments for these symptoms, such as tadalafil and testosterone replacement therapy, were administered, the symptoms did not improve. Here, we report a case of EjD associated with SV and prostate abscesses. Factors leading to SV atrophy are unknown; however, complications should be noted.

## Introduction

Seminal vesicle (SV) and prostatic abscesses are rare. Although the SV and prostate are highly involved in sexual function, little is known about the sexual dysfunction caused by these infectious diseases. The symptoms of these diseases include fever, dysuria, perineal pain, and other nonspecific symptoms [1,2]. There are several treatment options, including long-term conservative treatment with antibiotics and various methods of drainage, such as transperineal, transurethral, and transrectal [3,4], but no standardized treatment exists. Herein, we describe a case of SV atrophy and ejaculatory dysfunction (EjD) secondary to conservative therapy. No similar case reports were listed on PubMed as of April 4, 2025, using the search terms prostate, SV, and EjD. To the best of our knowledge, this case is believed to be the first reported case of SV atrophy and EjD following SV and prostatic abscesses.

## Case presentation

**Figure 1:**
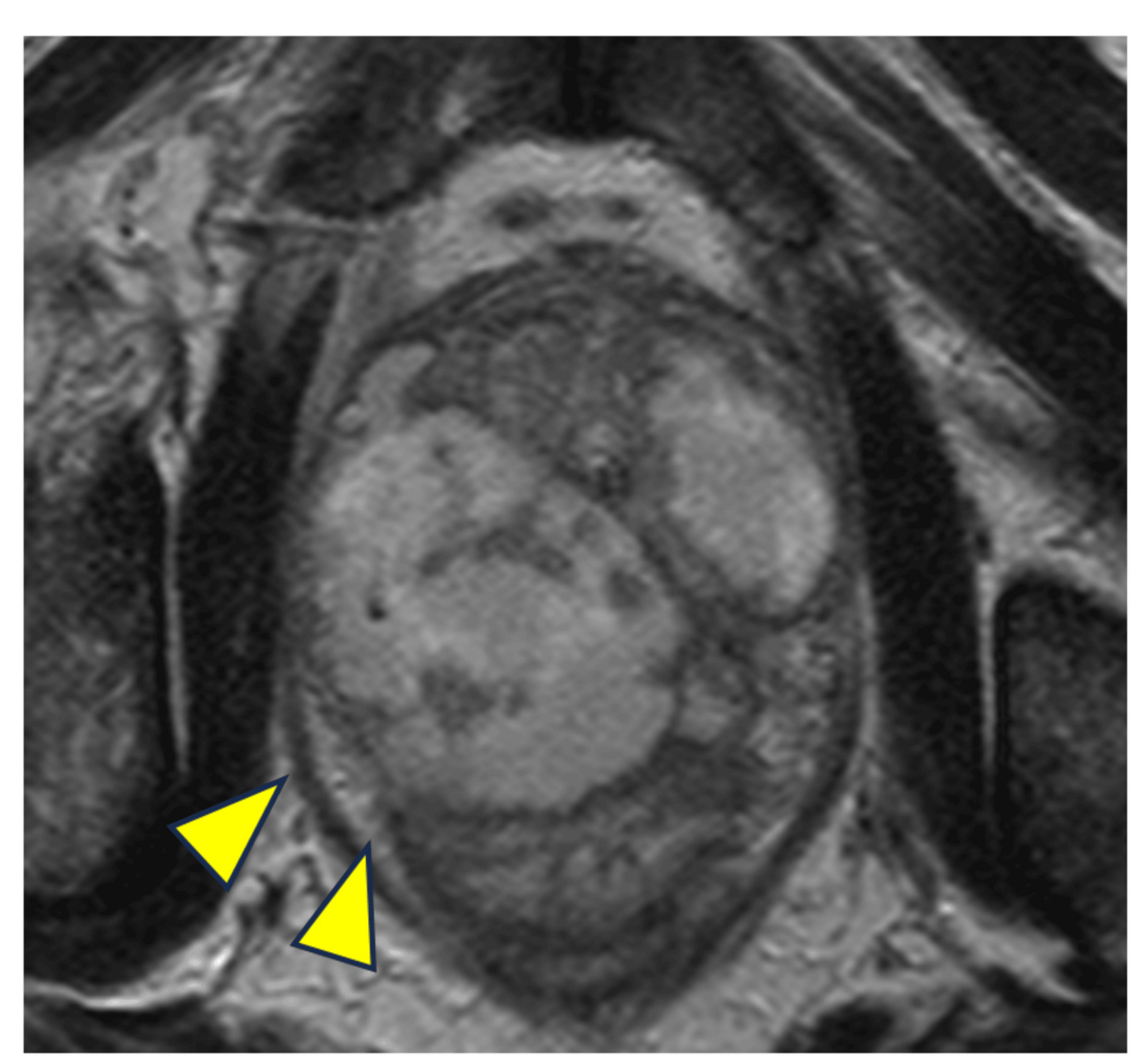
MRI of swollen prostate and seminal vesicles prior to initiation of treatment The prostate and bilateral seminal vesicles were markedly swollen, a finding that was suspicious for fluid collection in the arrowheads area.

A 56-year-old man visited a local clinic with fever and pain during urination. The patient was diagnosed with acute prostatitis based on an elevated inflammatory response and tenderness on rectal examination, and antibiotic therapy was initiated. There was no history of diabetes or any other special notes in the patient's medical history. First-line antibiotics did not improve inflammation, and magnetic resonance imaging (MRI) revealed an abscess involving the prostate and SVs (Figure 1). The type of antibiotic was changed, and the inflammation improved. Approximately one month after the end of treatment, EjD was observed, and the patient was referred to our hospital. The patient had an Aging Males' Symptoms score of 27 and an International Index of Erectile Function of 5 out of 10. Blood samples showed a negative inflammatory response and a mild decrease in the free testosterone level to 7.8 pg/mL (Table 1).

**Table 1:** Blood sample results at initial visit

Test	Measured value	Normal range
White blood cell	5500/μL	3300-8600/μL
Red blood cell	479 × 10^4^/μL	435-555 × 10^4^/μL
Hemoglobin	15.4 g/dL	13.7-16.8 g/dL
Platelet	19.9 × 10^4^/μL	15.8-34.8 × 10^4^/μL
C-reactive protein	0.02 mg/dL	< 0.14 mg/dL
Prostate-specific antigen	0.394 ng/mL	< 4 ng/mL
Luteinizing hormone	1.67 mIU/mL	0.79-5.72 mIU/mL
Follicle-stimulating hormone	10.8 mIU/mL	2-8.3 mIU/mL
Total testosterone	4.11 ng/mL	1.31-8.71 ng/mL
Free testosterone	7.8 pg/mL	4.6-19.6 pg/mL

**Figure 2:**
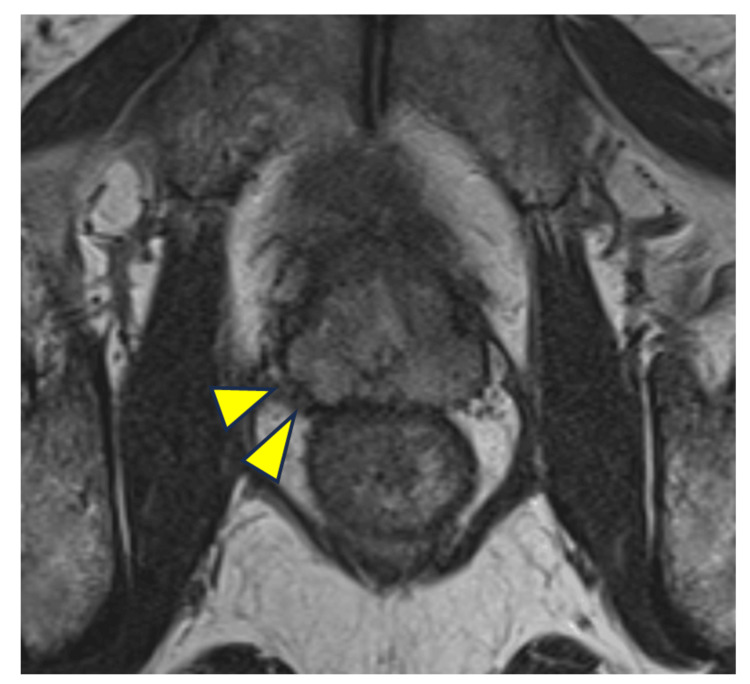
Prostate on MRI after conservative treatment Prostatic and bilateral seminal vesicle swellings improved and fluid collection almost disappeared (arrowheads).

**Figure 3:**
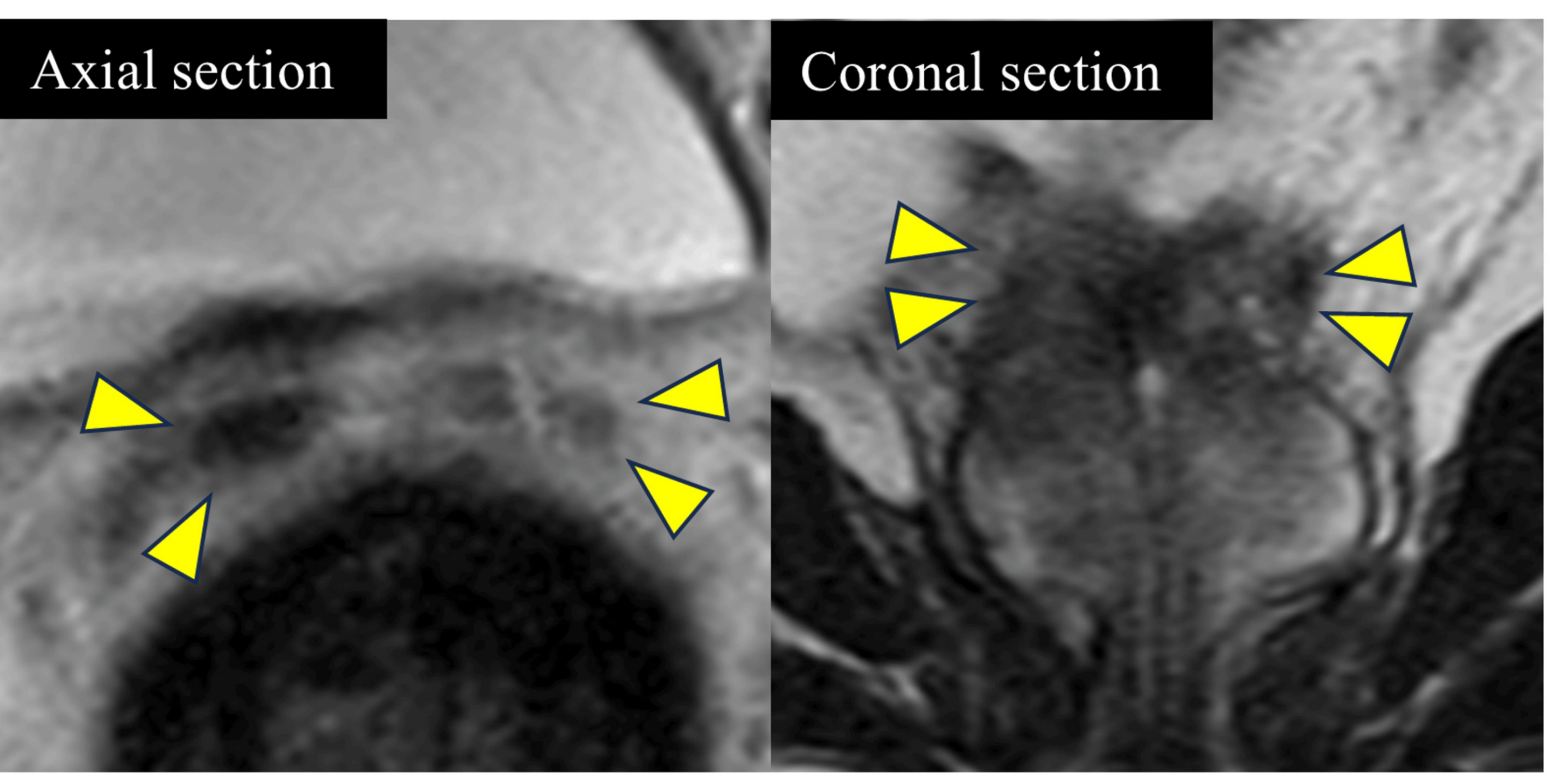
Atrophied seminal vesicles on MRI after conservative treatment No high-signal areas were observed within the bilateral seminal vesicles (indicated by arrowheads). The findings were suggestive of seminal vesicle atrophy and decreased seminal fluid.

MRI showed improvement in fluid collection in the prostate and SV, as well as severe atrophy of the SV (Figures 2-3). The patient complained of an inability to have an orgasm. Amoxapine and testosterone replacement therapy were administered to rule out retrograde ejaculation and erectile dysfunction; however, only improvement in erectile dysfunction was observed, and no improvement in EjD was achieved. Tadalafil and vitamin B did not improve the EjD. The patient was unable to have an orgasm, and no post-ejaculation urinalysis was performed. The patient was diagnosed with EjD other than retrograde ejaculation or psychogenic and classified as emissionless due to SV atrophy based on his medical history. However, the possibility could not be ruled out that it was EjD due to ejaculatory duct obstruction since the patient did not want to undergo an invasive contrast examination of the vas deferens. The patient did not wish to have children; therefore, the treatment was terminated, and the patient was followed up.

## Discussion

We encountered a case of SV atrophy and EjD secondary to the conservative treatment of SV and prostatic abscesses.

SV and prostatic abscesses are rare. Prostatic abscesses were reported to account for 0.5-2.5% of prostate disease, with an estimated incidence of 0.2-0.5% [5,6]. Since the prostate and SV are close, there have been reports of cases in which acute bacterial prostatitis spilled over to the SV and formed an abscess simultaneously, as in this case [7].

There is no standard treatment for SV or prostatic abscesses. Ludwig et al. reported the criteria for the treatment of prostatic abscesses with antibiotics alone to be within 1 cm in diameter [8]. If conservative treatment is not successful, transperineal, transrectal, or transurethral drainage may be performed with antibiotics [3,4]. In this case, the inflammatory response was relieved without drainage by antibiotics administered by the previous physician.

To the best of our knowledge, there are no reported cases of EjD with SV atrophy after an abscess. The SV is involved in semen production, and the vas deferens is a part of the seminal tract. The vas deferens also orifices into the prostate urethra. These factors may explain why SV and prostate abscesses can cause EjD. In the present case, because the patient was not aware of the orgasm, SV atrophy was considered the main cause of EjD. However, the patient did not want to undergo an invasive close examination, and the presence of obstruction to the passage of the vas deferens could not be evaluated. Therefore, we could not rule out the possibility that the patient had a coexisting ejaculatory duct obstruction due to inflammation. Because SV atrophy occurred after only one month, it was thought to be SV infarction associated with inflammation, although MRI could not point to any pelvic perfusion abnormality such as the SV artery. Because tadalafil has been reported to improve vascular endothelial function [9], the patient was administered tadalafil to improve blood flow; however, this did not lead to improvement. Amoxapine, a tricyclic antidepressant similar to imipramine, which has been reported to be effective for retrograde ejaculation [10], and testosterone replacement therapy were also administered concurrently to exclude the possibility of retrograde ejaculation or erectile dysfunction due to late-onset hypogonadism syndrome but were ineffective.

It is unclear whether conservative treatment led to SV atrophy, and more knowledge is required. In addition, treatment should be performed considering the possibility of SV atrophy, a secondary complication of SV abscesses that can cause ejaculation problems. Aggressive drainage may be considered for early inflammatory control, especially in young sexually active patients.

## Conclusions

Here, we report a case of SV atrophy and EjD associated with SV and a prostatic abscess. Although the factors leading to atrophy are unknown, early aggressive treatment should be considered, taking into account the presence of sexual activity and the hope of “raising a child,” since the abscess may affect sexual function. Post-treatment follow-up regarding complications such as this may also be important.
